# Evolution of flexible biting in hyperdiverse parasitoid wasps

**DOI:** 10.1098/rspb.2021.2086

**Published:** 2022-01-26

**Authors:** Thomas van de Kamp, István Mikó, Arnold H. Staniczek, Benjamin Eggs, Daria Bajerlein, Tomáš Faragó, Lea Hagelstein, Elias Hamann, Rebecca Spiecker, Tilo Baumbach, Petr Janšta, Lars Krogmann

**Affiliations:** ^1^ Institute for Photon Science and Synchrotron Radiation (IPS), Karlsruhe Institute of Technology (KIT), 76344 Eggenstein‐Leopoldshafen, Germany; ^2^ Laboratory for Applications of Synchrotron Radiation (LAS), Karlsruhe Institute of Technology (KIT), 76131 Karlsruhe, Germany; ^3^ Department of Biological Sciences, University of New Hampshire, Durham, NH 03824, USA; ^4^ Department of Entomology, State Museum of Natural History Stuttgart, 70191 Stuttgart, Germany; ^5^ Evolutionary Biology of Invertebrates, Institute of Evolution and Ecology, University of Tübingen, 72076 Tübingen, Germany; ^6^ Department of Animal Taxonomy and Ecology, Adam Mickiewicz University in Poznań, 61‐614 Poznań, Poland; ^7^ Department of Zoology, Faculty of Science, Charles University, 128 43 Prague 2, Czech Republic; ^8^ Institute of Biology, Systematic Entomology (190n), University of Hohenheim, 70593 Stuttgart, Germany

**Keywords:** mandibles, functional morphology, insect diversification

## Abstract

One key event in insect evolution was the development of mandibles with two joints, which allowed powerful biting but restricted their movement to a single degree of freedom. These mandibles define the Dicondylia, which constitute over 99% of all extant insect species. It was common doctrine that the dicondylic articulation of chewing mandibles remained unaltered for more than 400 million years. We report highly modified mandibles overcoming the restrictions of a single degree of freedom and hypothesize their major role in insect diversification. These mandibles are defining features of parasitoid chalcid wasps, one of the most species-rich lineages of insects. The shift from powerful chewing to precise cutting likely facilitated adaptations to parasitize hosts hidden in hard substrates, which pose challenges to the emerging wasps. We reveal a crucial step in insect evolution and highlight the importance of comprehensive studies even of putatively well-known systems.

## 1. Introduction

In terms of species numbers and morphological and ecological diversity, insects are by far the most diverse lineage of terrestrial organisms [1–3]. During more than 400 million years, insect mouthparts have evolved considerable modifications allowing the ecological diversification of biting/chewing, sucking or filtering lineages and contributing to the tremendous species richness of the group [4,5]. The mandibles of the earliest hexapod lineages (Collembola, Diplura and Protura) are characterized by a single posterior articulation allowing flexible movement along a ball-and-socket joint [6]. A major evolutionary step was the development of dicondylic mandibles with an additional anterior articulation to the head capsule [7], leading to the Dicondylia, which traditionally comprise all insects except the bristletails (Archaeognatha) [8]. Within Dicondylia, secondary monocondyly is known from insects, whose mouthparts are transformed into stylets (e.g. Hemiptera) [9], but all groups with chewing mouthparts are considered dicondylic. Dicondylic mandibles are generally linked to an increased biting force [10–12], which allowed insects to exploit new food sources [13]. A major consequence of this transformation was the loss of rotating motion of the mandibles and their confinement to movement in a single plane [12,14]. As a fixed axis of rotation requires fewer muscles to control mandibular movements, the complexity of mandibular musculature was gradually reduced from early hexapods to winged insects (Neoptera) [10]. In most derived Neoptera, mandibular movement is realized solely by two large antagonistic muscles, adductor and abductor. Both may be composed of several bundles of fibres [15,16] but insert at single attachment sites, often via sclerotized tendons.

The evolution of parasitoidism in Hymenoptera has led to one of the largest species radiations within insects [17–19]. Several morphological adaptations have been identified that triggered diversification processes during parasitoid evolution (e.g. wasp waist, venomous stinger) [20]. The role of mouthparts has mainly been studied in the context of feeding, but its role in emergence from host, mating behaviour, host handling and nest construction has also been discussed [21]. However, there were no hints that mouthpart evolution might have been a strong driver of parasitoid species radiations.

**Figure 1.**
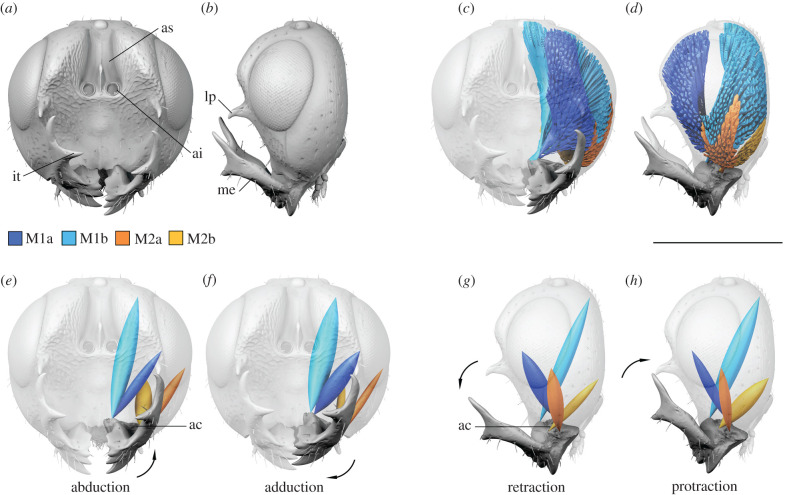
Head morphology and mandibular movement of Colotrechninae sp. (*a*) Head, frontal aspect. (*b*) Head, lateral aspect. (*c,d*) Original arrangement of the four mandibular muscles, M1a, M1b, M2a and M2b. (*e,f*) Putative biting movement. (*g,h*) Putative grasping movement. ac, anterior condyle; ai, antennal insertion; as, antennal scrobe; it, inner tooth; lp, lateral process; me, mandibular extension. Scale bar, 0.5 mm. (Online version in colour.)

In an undescribed species of parasitoid wasps, we discovered peculiar antler-like extensions on top of otherwise ordinary-looking chewing mandibles (figure 1). These extensions correspond to forward-projecting processes on the face and potentially serve as a grasping tool. Both grasping and chewing obviously cannot be realized by dicondylic mandibular movement. We analysed the functional morphology of the mandibles of this extraordinary specimen by synchrotron X-ray microtomography and found evidence for flexible mandibular movement, contradicting the current hypothesis of largely conserved mandibular articulations and musculature in chewing insects. The undescribed species belongs to the superfamily Chalcidoidea, which comprises one of the largest groups of insects with an estimated 500 000 predominantly parasitoid species [22]. Until now there was no convincing hypothesis which morphological features might have facilitated their unparalleled diversification [23,24].

To test whether flexible mandibular movement represents a singular evolutionary event or might play a larger role in parasitoid evolution, we analysed the occurrence of this type of mandible throughout Chalcidoidea and all major lineages of Hymenoptera and correlated the morphological characters with the most recent molecular phylogenies of Hymenoptera [25] and Chalcidoidea [24].

## 2. Results

### (a) Mandibular morphology of Colotrechninae sp.

The face of Colotrechninae sp. is excavated and bears a pair of lateral facial processes next to the inner eye margins, which are each flanked by a single elongate seta. These processes are pointed ventrally and are slightly curved inwards. They are situated slightly below the level of the antennal insertions. The antennae are inserted high on the face within deep antennal scrobes. The mandibles possess five teeth each. Their outer surfaces feature conspicuous, distally pointed extensions, reaching distally to the lateral facial processes. Further, each mandibular extension carries a distinct inner tooth (figure 1*a,b*).

The mandible is loosely articulated to the head capsule by a single anterior condyle. A posterior condyle is completely absent. All mandibular muscles insert directly to the mandible and not via sclerotized tendons (figure 1*c,d*). Two separate pairs of muscle bundles are developed and connect the mandible to the cranium. One pair (M1) has its anterior bundle (M1a) originating from the frons and its posterior bundle (M1b) from the gena. Both bundles insert at the inner angle of the mandibular base. The second pair (M2) has its anterior (M2a) and posterior bundles (M2b) originating at the ventral part of the gena and inserting to flanges of the outer margin of the mandibular base (figure 1*c,d*).

### (b) Mandibular character distribution throughout Hymenoptera

**Figure 2.**
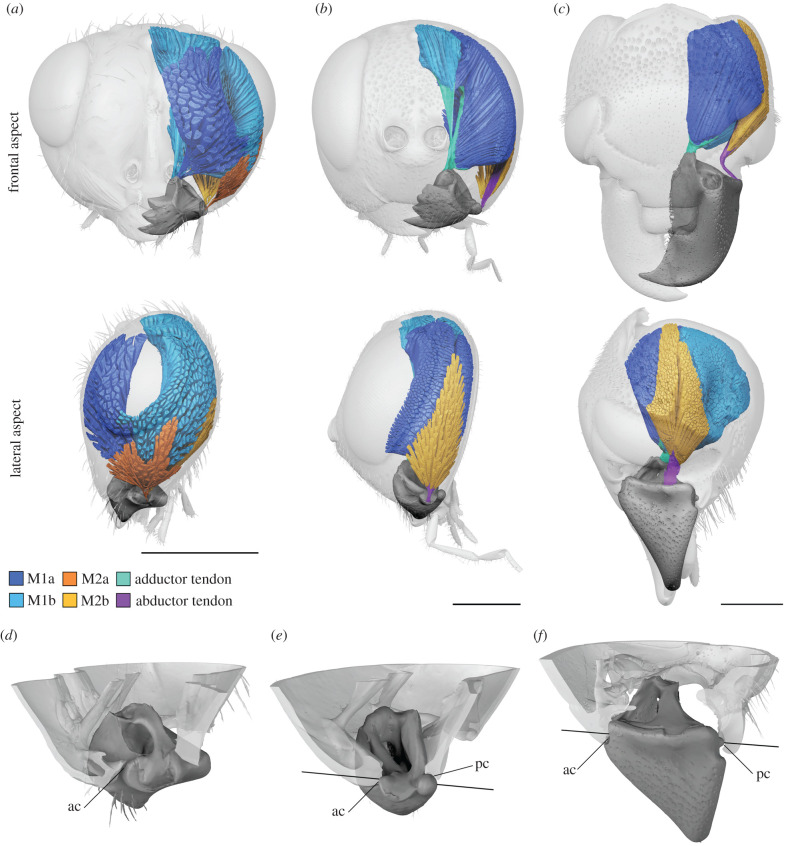
Comparison of mandibles and attached musculature in two wasps and a histerid beetle. (*a*) *Chromeurytoma* (Chalcidoidea). (*b*) *Zeuxevania* (Evanioidea). (*c*) *Margarinotus* (Coleoptera: Hydrophiloidea). (*d*–*f*) Mandibular articulations in the respective species, lateral view, cranium cut. (*d*) Monocondylic mandible with a single anterior articulation. (*e*,*f*) Dicondylic mandibles with anterior and posterior articulations that restrict mandibular movement to a fixed axis of rotation (indicated). ac, anterior condyle; pc, posterior condyle. Scale bars, 0.5 mm. (Online version in colour.)

**Figure 3.**
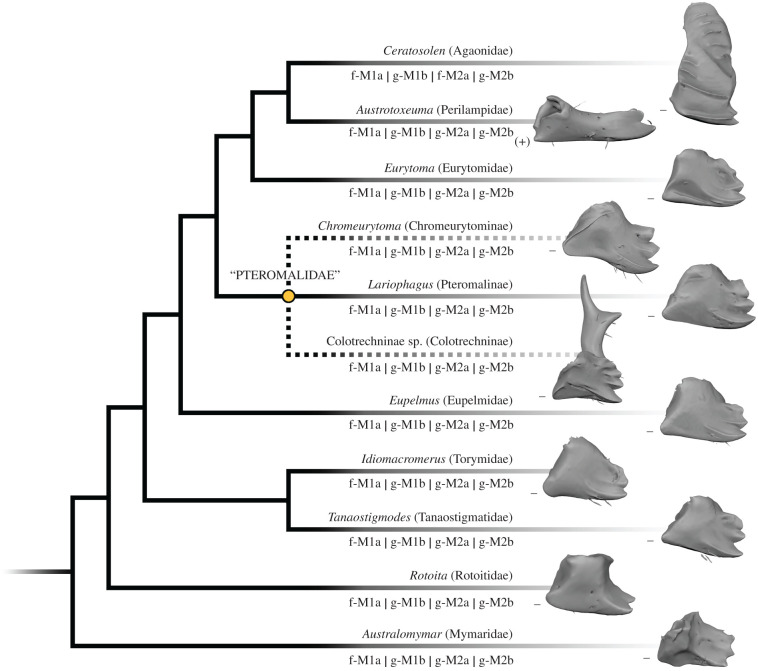
Characters of mandibles and mandibular musculature found in the examined taxa mapped on the molecular phylogeny of Chalcidoidea from Peters *et al*. [24]. The placement of *Rotoita* follows Heraty *et al*. [23]. Dotted lines indicate taxa of uncertain phylogenetic position. f- , originates from the frons; g-, originates from the gena; M1a, M1b, M2a, M2b, mandibular muscle bundles; (+), posterior condyle indicated; –, posterior condyle reduced.

**Figure 4.**
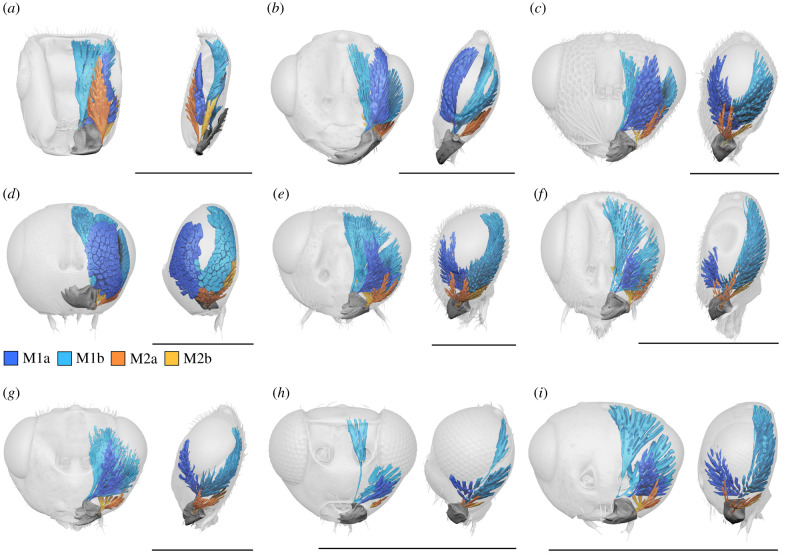
Mandibles and attached musculature throughout Chalcidoidea. (*a*) *Ceratosolen* (Agaonidae). (*b*) *Austrotoxeuma* (Perilampidae). (*c*) *Eurytoma* (Eurytomidae). (*d*) *Lariophagus* (Pteromalidae). (*e*) *Eupelmus* (Eupelmidae). (*f*) *Idiomacromerus* (Torymidae). (*g*) *Tanaostigmodes* (Tanaostigmatidae). (*h*) *Australomymar* (Mymaridae). (*i*) *Rotoita* (Rotoitidae). Scale bars, 0.5 mm. (Online version in colour.)

Despite huge variation in overall mandibular shape, all other Chalcidoidea examined (figure 2*a,d*, 3 and 4) share the monocondylous condition found in Colotrechninae sp. Only in *Austrotoxeuma*, a posterior condyle is slightly indicated but not articulated to the head capsule. The mandibular musculature of all other Chalcidoidea is also characterized by two muscles (M1 and M2) with two bundles each, which individually insert on the mandible. As in Colotrechninae sp., M1a originates always from the frons and M1b from the gena. In most Chalcidoidea, M2a and M2b originate from the ventral gena. A notable exception is the flattened head of *Ceratosolen* (Agaonidae), where M2a originates from the frons.

**Figure 5.**
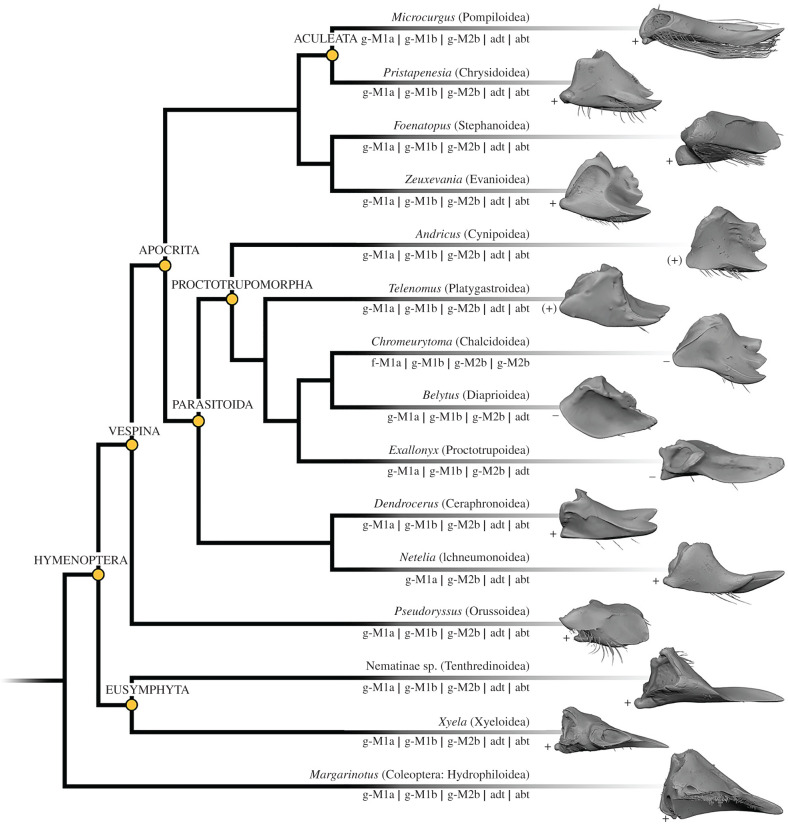
Characters of mandibles and mandibular musculature found in the examined taxa mapped on a molecular phylogeny of Hymenoptera [25]. abt, abductor tendon; adt, adductor tendon; f-, originates from the frons; g-, originates from the gena; M1a, M1b, M2a, M2b, mandibular muscle bundles; +, posterior condyle distinct; (+), posterior condyle indicated; –, posterior condyle reduced.

Regarding mandibular morphology, we also found a reduction of the posterior condyle in other Proctotrupomorpha, while it was distinct in all other groups (figures 2*e* and 5). In close relatives of Chalcidoidea (*Belytus* (Diaprioidea) and *Exallonyx* (Proctotrupoidea)), the posterior condyle is completely reduced (no posterior articulation with the head capsule), while it is indicated but without form closure around the condyle in the more distantly related lineages (*Telenomus* (Platygastroidea) and *Andricus* (Cynipoidea)).

**Figure 6.**
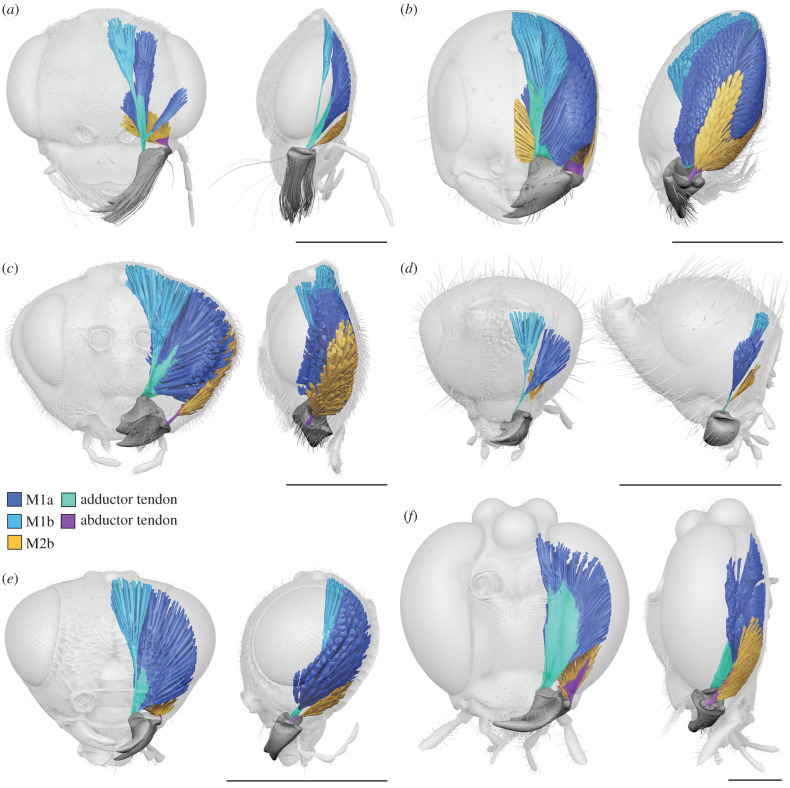
Mandibles and attached musculature throughout Hymenoptera. (*a*) *Microcurgus* (Pompiloidea). (*b*) *Pristapenesia* (Chrysidoidea). (*c*) *Andricus* (Cynipoidea). (*d*) *Belytus* (Diaprioidea). (*e*) *Dendrocerus* (Ceraphronoidea). (*f*) *Netelia* (Ichneumonoidea). Scale bars, 0.5 mm. (Online version in colour.)

The mandibles in all hymenopteran lineages except Chalcidoidea have single insertion points for M1 and M2 (figures 2*b* and 6) and muscle bundles usually insert via sclerotized tendons (abductor tendon not recognizable only in *Belytus* (Diaprioidea) and *Exallonyx* (Proctotrupoidea)). With the exception of *Netelia* (Ichneumonoidea), the tendons of M1 are split distally and attach to separate muscle bundles. Both muscles (M1 and M2) originate from the gena. This largely corresponds to the condition found in other mandibulate insects, such as beetles (figure 2*c,e*).

## 3. Discussion

### (a) Functional interpretation

In Chalcidoidea, the mandible and its articulation as well as the associated musculature are highly modified compared to other pterygote insects, with fundamental functional consequences. The mandible is articulated to the head capsule by just a single anterior condyle, instead of two condyles as generally postulated for pterygote insects with biting mouthparts. This abolishes a functional restriction of mandibular movement to a single plane. Instead, in combination with highly modified mandibular musculature, a flexible movement of mandibles can be achieved, including adduction, abduction, protraction, retraction, rotation and any combination of these movements. In Chalcidoidea, M1 is therefore not restricted in its function as an adductor and M2 not as abductor as in other pterygote insects. By contrast to all other groups examined, M1a originates from the frons (figures 1, 2*a* and 4), instead of the gena (figures 2*b,c* and 6). This allows the mandible to be pulled from an anterior direction, supporting mandibular movement along multiple planes. Moreover, each bundle of M1 and M2 inserts independently at the mandible, whereas in other biting insects, these bundles insert via a single sclerotized tendon [26] (figures 2*b,c* and 6). Based on the observations mentioned above we conclude that in the mandibular musculature of Chalcidoidea each muscle bundle acts as a functionally separate entity. Standard biting can still be achieved by the antagonizing bundles of M1 and M2. In this case, these pairs of muscle bundles would act as adductor (M1) and abductor (M2) (figure 1*e,f*). By contrast, flexible mandibular movement along multiple degrees of freedom is realized by the interplay of all four muscle bundles acting independently. For upward-directed movement of mandibles, the two posterior bundles M1b and M2b would act as protractors and their anterior counterparts M1a and M2a as retractors. In Colotrechninae sp., this movement allows for a closure between the tips of the mandibular antlers and the lateral facial processes (figure 1*g,h*; see electronic supplementary material, movie S1).

### (b) Evolutionary considerations

Chalcidoidea are unique among parasitoids in targeting the largest diversity of host taxa and in exhibiting the largest number of feeding types defined for parasitoid wasps [27]. However, unlike other parasitoid wasp groups, such as Ichneumonoidea, Chalcidoidea do not often develop on free-living hosts, such as ectophytophagous larvae of butterflies, moths or beetles. A large majority of chalcid species develop on enclosed host stages with reduced mobility. Examples include wood and stem borers, leaf-miners or inhabitants of galls, seeds and fruits [27]. Interestingly, most of these host associations are displayed by ectoparasitoid chalcids, which enables the parasitoid larvae to develop within the protection of a concealed environment without being exposed to the host immune system, thereby combining advantages of endo- and ectoparasitoid lifestyles. A consequence of this strategy is the challenge of the freshly emerged wasp to escape from the concealed environment, which is usually achieved by time-consuming biting through the surrounding substrate. In this respect, the host biology of *Lariophagus distinguendus* (Förster, 1841) (Pteromalidae) is typical for the majority of chalcid wasp species. Flexible mandibular movements during its host eclosion are clearly visible (see electronic supplementary material, movie S2): both mandibles can move independently at the same time. This allows precise cutting, as the mandibles can operate under different angles to the substrate and to each other. This flexible movement might be especially helpful in an environment with spatial constraints, where force has to be applied with minimal movements of the head itself. These constraints can either be caused by arthropod host eggs, as in the earliest chalcid lineage Mymaridae, or by the substrate surrounding parasitoids emerging from their enclosed hosts, as in the majority of Chalcidoidea. Therefore, we assume that flexible mandibular movement played an important role in the evolution of diverse host associations.

The flexible articulation of the mandibles represents a modification unique among insects. Close relatives of Chalcidoidea (Diaprioidea and Proctotrupoidea) already show at least a partial reduction of the posterior condyle (figure 5), which may be interpreted as an intermediate state putatively leading to increased flexibility. In a second step, the complete reduction of the posterior condyle is accompanied by modified musculature with a functional separation, different origins and insertions of abductors and adductors. This resulted in full flexibility of mandibles in Chalcidoidea. Interestingly, this case of secondary monocondyly is realized differently than in the primarily monocondylic hexapods (Collembola, Diplura and Protura). By contrast to the latter, only the anterior (secondary) articulation remained to facilitate mandibular movements in Chalcidoidea.

The bizarre mandibles in Colotrechninae sp. represent a unique evolutionary step that was facilitated by their flexible articulation. The antler-like extensions of the mandibles can interlock with the lateral facial processes. In combination with the excavated face, this strongly hints to a grasping mechanism, while the standard biting function of the mandibles can be maintained (figure 1*e–h*, electronic supplementary material, movie S1). A potential grasping mechanism could be used for clasping the hosts prior to oviposition. Currently, the host biology of Colotrechninae sp. is obscure and the new species is only known from a single female specimen. The face and mandibular morphology of Colotrechninae sp. is unparalleled among extant insects but shows a staggering similarity to the ‘hell ants’ (Formicidae: Haidomyrmecinae) described from Cretaceous amber deposits [28]. ‘Hell ants’ were able to move their mandibles vertically to interlock with a cephalic projection, and a function as prey-capturing device has been verified based on the discovery of a fossil specimen holding its roach-like prey [29]. The mandibular articulation of ‘hell ants’ is currently not known but the observed similarities to Colotrechninae sp. are undoubtedly the result of convergent evolution. Another similarity between Colotrechninae sp. and ‘hell ants’ is the presence of sensory organs close to the cephalic projection. In Colotrechninae sp., a single seta is situated close to each of the paired facial processes. In ‘hell ants’, the setae are more prominent and situated in a row along the outer margin of the cephalic projection. In both cases, these setae might have triggered the (potential) grasping mechanism.

### (c) Mouthpart evolution triggers diversification of arthropods

The mandible of Chalcidoidea represents an evolutionary novelty that likely played an important role during an extremely large insect radiation process leading to the estimated 500 000 species of this superfamily. It has long been known that the evolution of mandibles in the Mandibulata (the most speciose group of Arthropoda comprising millipedes, crustaceans and hexapods) and its modifications in the dicondylic insects have triggered large species radiations [13]. Our results suggest that the secondary reversal to monocondylic mandibles in Chalcidoidea (this time affecting the posterior condyle instead of the anterior typical for monocondylic hexapods) had further dramatic evolutionary consequences for parasitoids and helped them to exploit novel host systems, leading to complex niche differentiations and adaptive radiations.

## 4. Methods

### (a) Taxon sampling

Representative taxa of ethanol-preserved Hymenoptera and one species of Coleoptera (electronic supplementary material, table S1) have been selected and studied and voucher specimens are deposited at the State Museum of Natural History, Stuttgart.

### (b) Synchrotron X-ray microtomography

Tomographic scans of ethanol-preserved insect heads were performed at the UFO-I station of the Imaging Cluster at the KIT light source using a parallel polychromatic X-ray beam produced by a 1.5T bending magnet. The beam was spectrally filtered by 0.5 mm aluminium and the resulting spectrum had a peak at about 15 keV, with a full-width at a half maximum bandwidth of about 10 keV. A fast indirect detector system was employed, consisting of a 12 µm LSO:Tb scintillator [30] and a diffraction-limited optical microscope (Optique Peter) [31] coupled with a 12bit pco.dimax high speed camera with 2016 × 2016 pixels. Scans were done by taking 3000 projections at 70 fps and an optical magnification of 10×, resulting in an effective pixel size of 1.22 µm. We used the control system concert [32] for automated data acquisition and online reconstruction of tomographic slices for data quality assurance. Online and final data processing included flat field correction and phase retrieval of the projections based on the transport of intensity equation [33]. X-ray beam parameters for algorithms in the data processing pipeline were computed by *syris* [34] and the execution of the pipelines, including tomographic reconstruction, was performed by the UFO framework [35].

### (c) Post-processing of tomographic data

Tomographic slices were converted to 8 bit and cropped to the region of interest. In Amira 5.6. heads, mandibles and mandibular muscles were pre-segmented in the software's segmentation editor. The labels served as input for automated segmentation, which was performed using the online platform Biomedisa (https://biomedisa.org) [36]. Segmentation results were again imported into Amira 5.6 and minor errors were corrected. The final labels were converted into polygon meshes, exported as OBJ files and reassembled and smoothed in CINEMA 4D R20.

### (d) High-resolution videography

The specimens of *L. distinguendus* used in this study originate from the laboratory colonies of the Biologische Beratung GmbH (Berlin), where they were bred on larvae of *Sitophilus oryzae* (Linnaeus, 1763) (Coleoptera: Curculionidae) that developed in grains of the common wheat *Triticum aestivum* L. The infested wheat grains were observed and the hatching wasps were recorded using a Nikon DSC D90 camera mounted on a Leica MZ 12.5 stereomicroscope.

## Data accessibility

The tomographic volumes examined in this study are available from the Dryad Digital Repository: https://doi.org/10.5061/dryad.0rxwdbs1x [37].
